# DAS28 best reflects the physician's clinical judgment of response to infliximab therapy in rheumatoid arthritis patients: validation of the DAS28 score in patients under infliximab treatment

**DOI:** 10.1186/ar1787

**Published:** 2005-07-08

**Authors:** Bert Vander Cruyssen, Stijn Van Looy, Bart Wyns, Rene Westhovens, Patrick Durez, Filip Van den Bosch, Eric M Veys, Herman Mielants, Luc De Clerck, Anne Peretz, Michel Malaise, Leon Verbruggen, Nathan Vastesaeger, Anja Geldhof, Luc Boullart, Filip De Keyser

**Affiliations:** 1 Department of Rheumatology, Ghent University Hospital, Belgium; 2 Department of Electrical Energy, systems and automation, Ghent University, Gent, Belgium; 3 Department of Rheumatology, University Hospitals KULeuven, Leuven, Belgium; 4 Department of Rheumatology, Cliniques Universitaires Saint-Luc, Brussels, Belgium; 5 Department of Rheumatology, University Hospital Antwerp, Antwerp, Belgium; 6 Department of Rheumatology, University Hospital Brugmann, Brussels, Belgium; 7 Department of Rheumatology, University Hospital Liège, Liège, Belgium; 8 Department of Rheumatology, AZ Vrije Universiteit Brussel, Brussels, Belgium; 9 Department of Medical affairs, Schering Plough, Brussels, Belgium; 10 Department of Medical affairs, Centocor, Leiden, the Netherlands

## Abstract

This study is based on an expanded access program in which 511 patients suffering from active refractory rheumatoid arthritis (RA) were treated with intravenous infusions of infliximab (3 mg/kg+methotrexate (MTX)) at weeks 0, 2, 6 and every 8 weeks thereafter. At week 22, 474 patients were still in follow-up, of whom 102 (21.5%), who were not optimally responding to treatment, received a dose increase from week 30 onward. We aimed to build a model to discriminate the decision to give a dose increase. This decision was based on the treating rheumatologist's clinical judgment and therefore can be considered as a clinical measure of insufficient response. Different single and composite measures at weeks 0, 6, 14 and 22, and their differences over time were taken into account for the model building. Ranking of the continuous variables based on areas under the curve of receiver-operating characteristic (ROC) curve analysis, displayed the momentary DAS28 (Disease Activity Score including a 28-joint count) as the most important discriminating variable. Subsequently, we proved that the response scores and the changes over time were less important than the momentary evaluations to discriminate the physician's decision. The final model we thus obtained was a model with only slightly better discriminative characteristics than the DAS28. Finally, we fitted a discriminant function using the single variables of the DAS28. This displayed similar scores and coefficients as the DAS28. In conclusion, we evaluated different variables and models to discriminate the treating rheumatologist's decision to increase the dose of infliximab (+MTX), which indicates an insufficient response to infliximab at 3 mg/kg in patients with RA. We proved that the momentary DAS28 score correlates best with this decision and demonstrated the robustness of the score and the coefficients of the DAS28 in a cohort of RA patients under infliximab therapy.

## Introduction

Rheumatoid arthritis (RA) is a complex disease with a broad spectrum of manifestations that requires an early intensive therapy in order to avoid joint destruction and physical disability. In order to measure the effect of therapy in daily practice and in clinical trials, many variables are recorded and different composite indices have been proposed to measure the remaining disease activity or the response to treatment. Those variables may cover items such as patient self-reported questionnaires, physician's scores including different joint scores, and serum markers of systemic inflammation.

Infliximab, in combination with methotrexate (MTX), is a highly effective therapy for a majority of RA patients. After an induction scheme at weeks 0, 2 and 6, the indicated dose of this therapy is 3 mg/kg every 8 weeks, although the ATTRACT trial suggested that a higher dose of 10 mg/kg every 8 weeks or a shorter perfusion interval may add benefit [1-3].

The present study is based on an expanded-access program in which patients suffering from active refractory RA were treated with intravenous infusions of infliximab (3 mg/kg + MTX) at weeks 0, 2, 6 and every 8 weeks thereafter. At week 22, patients not optimally responding to treatment could receive a dose increase of 100 mg (1 vial) per infusion from week 30 onwards [4]. The effect of dose escalation for the patients of this cohort has been discussed previously [4]. The decision to increase the dose was based on the treating rheumatologist's clinical judgment and can be considered as a measure of insufficient response to infliximab. It might be questioned which variables can be measured to best evaluate the effect of therapy and remaining disease activity in daily practice (and in clinical trials). The aim of the present analyses was to evaluate whether the decision to increase the dose could be reflected by using single variables or composite indices, alone or together in a model. We also wanted to evaluate whether this decision was mainly based on differences over time or on momentary disease activity.

## Methods

### Study population

A total of 511 patients, suffering from active refractory RA [5], were treated with intravenous infusions of infliximab (3 mg/kg) at weeks 0, 2, 6 and every 8 weeks thereafter in combination with MTX (a minimal dose of 15 mg/kg was recommended). Between week 0 and week 22, 37 patients dropped out for the following reasons: 16 patients stopped due to side effects (four infusion reactions, five infections, one malignancy, one pancytopenia, five disease-related complications), 12 patients stopped for withdrawal of consent and 9 patients stopped for protocol violation. Of the remaining 474 patients, 102 (22%) patients, who were not optimally responding to treatment according to the treating rheumatologist's opinion, received a dose increase of 100 mg (1 vial) per infusion from week 30 on. Throughout the first 22 weeks, dosage of MTX, steroids and non-steroidal anti-inflammatory drugs remained unchanged.

### Evaluated variables

When designing the model, we took the following single variables into account at weeks 0, 6, 14 and 22: 28 and 66/68 swollen/tender joint counts, erythrocyte sedimentation rate (ESR; mm/h), C-reactive protein (CRP; mg/l), Health Assessment Questionnaire (HAQ; 0–3), physician's global assessment of disease activity (visual analogue scale (VAS); 0–100 mm), patient's global assessment of disease activity (VAS 0–100 mm), patient's assessment of pain (VAS 0–100 mm), patient's assessment of fatigue (VAS 0–100 mm) and all subscales of the SF-36 questionnaire (0–100 points) [6]. DAS28 (Disease Activity Score including a 28-joint count) [7] and other composite scores such as simplified disease activity index (SDAI), clinical disease activity index (CDAI) [8,9] and the alternative DAS28 scores [10,11] (Table 1) were calculated after data collection so that the treating rheumatologist was unaware of the exact values of those composite scores. Also, differences over time and the DAS28 response (no, moderate or good) and the ACR (American College of Rheumatology) response (no/20/50) were computed [12,13].

### Statistics

We opted to use only statistical methods that are available in a classical statistical package (SPSS 12.0; SPSS, Inc, Chicago, IL, USA) or could be computed manually. When needed, the continuous variables were normalized (by taking the square root of the joint counts and the natural logarithm of CRP and ESR). Robustness of the discriminant analyses and logistic regressions was confirmed by the use of a random train and test set. Missing values were handled by pairwise complete case analysis. This means that a case with no missing values for a group of variables is included in the analysis of that group of variables. The case may have missing values for variables used in other analyses. Confidence intervals (95% CI) for sensitivity or specificity were calculated based on the method proposed by Harper [14]. The areas under the curves (AUCs) of receiver operating characteristic (ROC) curves were calculated. A higher AUC indicates that a single variable has better discriminative characteristics. A statistical test to compare AUCs of two variables tested on the same population has been described by Hanley [15]. Continuous and categorical variables were compared by adapting the cut-off of the continuous variables to the same specificity level as the categorical variable so that sensitivities could be evaluated and compared [16]. The selection and comparison of variables by curve analysis was performed since this method gives a valid ranking of variables and does not (in contrast to ranking methods based on *p *values) depend on the number of subjects available for that specific variable [17]. In order to find the true maximal model and to avoid sticking at a local maximal model, we used different strategies for the construction of the final model: binary logistic regressions and discriminant analyses were performed with the default options of SPSS 12.0 and stepwise construction of models was performed by conditional forward and backward elimination for logistic regression and by Wilk's lambda for discriminant analysis using the strategy described by Hosmer and Lemeshow [18].

### Ethics

All patients signed informed consent. This study was approved by the local ethics committees.

## Results

### Ranking of continuous variables

In order to select the most important variables that correlate with the decision to give a dose increase at week 22, we calculated the AUC of ROC curve analysis for all continuous variables and ranked them based on this AUC [17]. Since crossing over of ROC curves may affect the diagnostic properties of a variable without changing the AUC, we also ranked the variables based on sensitivity levels by adapting the cut-off to a given preset specificity level of 95% [16].

Both ranking methods displayed that the DAS28 score at week 22 had the highest ability to discriminate the physician's decision to give a dose increase. Table 2 displays the 10 most important variables ranked by AUC of ROC curve analysis and by the sensitivity at the 95% specificity level. Using the method described by Hanley [15], we found that there was a significant difference in AUC between the two first ranked parameters: DAS28 at week 22 and the 28 tender joint count at week 22 (AUC = 0.840 versus 0.797, *p *= 0.02). Additionally, most variables were ranked in such a way that each variable was represented first by its measure at week 22 before it was represented by a measure at another week.

### Evaluation of the response scores

To evaluate categorical scores, we adapted the cut-off of the variable with the highest ranking (DAS28 at week 22) to the specificity of the categorical score and compared the sensitivities [16]. For the decision to give a dose increase, ACR response not reaching the ACR20 criterion ('no ACR response') had a sensitivity of 69.6% (95% CI: 65.2–74.0) and a specificity of 64.2% (95% CI: 59.6–68.8). When we adapted the cut-off of the DAS28 at week 22 to a specificity of 64.2% (DAS28 = 4.01), we obtained a sensitivity of 80.0% (95% CI: 75.2–84.7). 'No DAS28 response' had a sensitivity of 46.7% (95% CI: 40.8–52.6) and a specificity of 83.3% (95% CI: 78.9–87.7). When we adapted the cut-off of the DAS28 to a specificity of 83.3% (DAS28 = 4.77), we obtained a sensitivity of 67.5% (95% CI: 61.9–73.1). Similar results were obtained when looking at the ACR50 and the good DAS28 response criterion (Table 3).

Additionally, we fitted a logistic regression model with the decision to give a dose increase as a dependent variable and DAS28 at week 22, DAS28 response and ACR response as categorical covariates. These analyses retained DAS28 at week 22 as the only significant covariate in the model (data not shown).

### Effects of change of scores over time on the physician's decision

**Figure 1:**
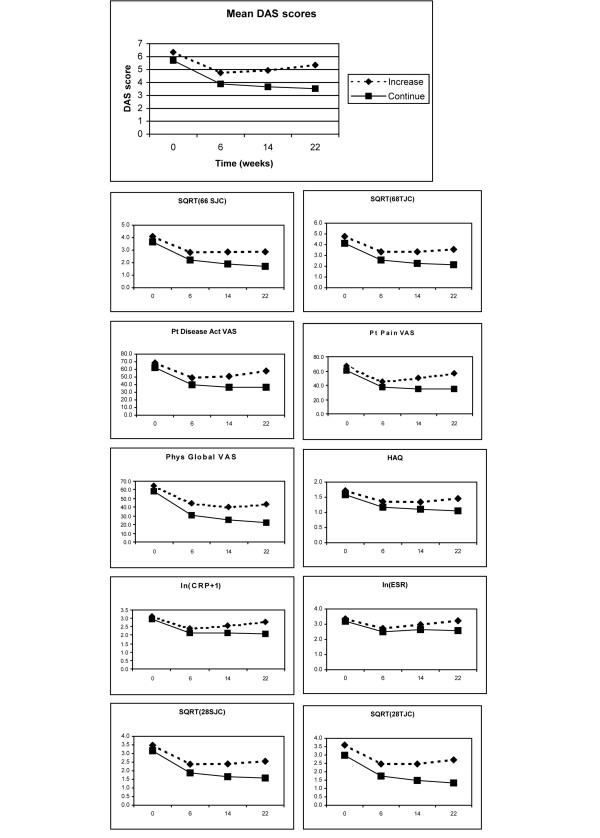
Plot of the mean scores over time. Act, activity; ESR, erythrocyte sedimentation rate; HAQ, Health Assessment Questionnaire; SJC, swollen joint count; TJC, tender joint count; pt, patient; Phys, physician; SQRT, variable normalized by taking the squared root; ln, variable normalized by taking the natural logarithm; VAS, visual analogue scale.

To evaluate the effect of differences over time, we plotted the means of the most important normalized continuous variables over time (Fig. 1). The plot of the variable with the highest ranking (DAS28) shows that patients who get a dose increase have a (significantly) higher disease activity at baseline and, after an initial decrease of disease activity, regain disease activity from week 6 on. To evaluate this, we calculated differences in DAS28 scores between baseline and week 22 (delta DAS28 0–22), and between week 6 and week 22 (delta DAS28 6–22). Indeed, patients who get a dose increase regain some disease activity between week 6 and week 22 (mean delta DAS28 6–22: -0.4 versus +0.4, *p *< 0.001), which is reflected in a smaller decrease of disease activity between baseline, and week 22 (mean delta DAS28 0-22: -2 versus -1, *p *< 0.001). However, the AUC of the ROC curve of delta DAS28 0–22 was 0.725 (95% CI: 0.659–0.790) and the AUC for delta DAS28 6–22 was 0.672 (95% CI: 0.590–0.754), which is much lower than the AUC of the momentary DAS28 (0.840) at week 22. Additionally, when we fitted a logistic regression model with the decision to give a dose increase as a dependent variable and DAS28 at week 22, delta DAS28 0–22 and delta DAS28 6–22 as covariates, only DAS28 at week 22 was a significant variable in the model.

Similar analyses were performed for the other variables. The AUC of the differences between weeks 0–22, weeks 6–22 and weeks 14–22 of the other variables were all less than 0.700 (data not shown). These analyses indicate that, although the differences in disease activity over time are statistically significant, those differences over time are not important enough to incorporate in a model to discriminate the physician's decision.

### Building a model to discriminate the physician's decision to give a dose increase

The first three analyses (ranking of continuous variables, evaluation of the response scores and effects of change of scores over time on the physician's decision) allowed us to narrow the selection of variables for the model by eliminating variables that are already incorporated into the DAS28 (or are highly related to them such as CRP and 68 tender joint and 66 swollen joint count) and taking into account only those variables at week 22. This resulted in the following list: DAS28, HAQ, physician global VAS, patient pain VAS, patient fatigue VAS and the scores of the SF36 questionnaire at week 22. We screened those variables using forward and backward elimination in a logistic regression model and by the stepwise Wilk's lambda method. The probability scores of the logistic regression and discriminant scores we thus obtained were compared using ROC curve analysis. The model with the highest AUC was a model from discriminant analysis with the following variables (and standardized canonical discriminant function coefficients): DAS28 week 22 (0.863), physician global VAS (0.796), patient pain VAS (0.735), and physical functioning (-0.227). The discriminant score of this model had an AUC of 0.870 (95% CI: 0.828–0.912) with a sensitivity at the 95% specificity level of 45.5% (95% CI: 38.7–50.3).

### Evaluation of the discriminant score of the variables of DAS28

**Figure 2:**
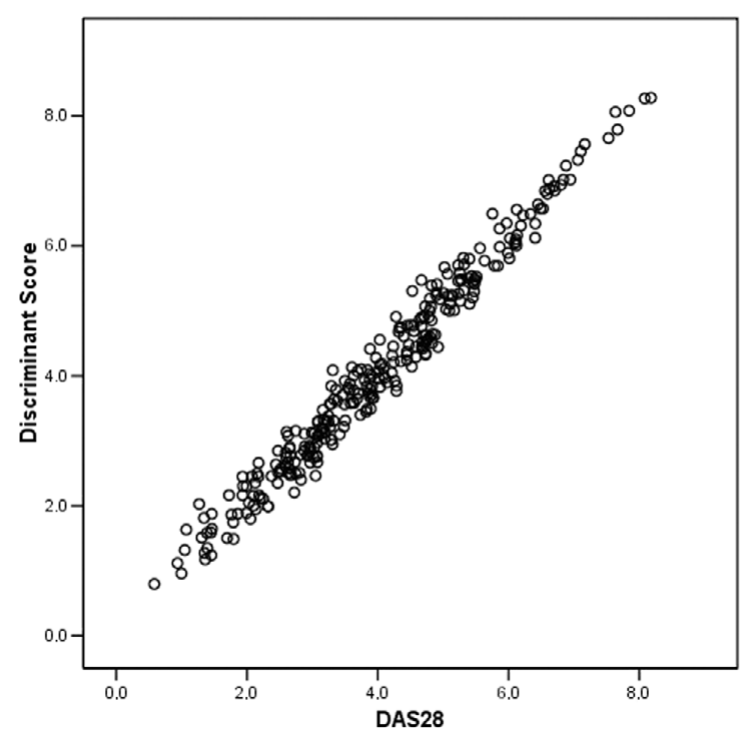
Validation of the DAS28 score and coefficients (see text). ESR, erythrocyte sedimentation rate; VAS, visual analogue scale.

To validate the score and coefficients of the DAS28, we calculated a discriminant function using the (normalized) variables of the DAS28 score: 28 tender and swollen joint count, ESR and patient global VAS. After rescaling, we obtained the following discriminant coefficients: 0.52 for 28 tender joint count (28TJC), 0.28 for 28 swollen joint count (28SJC), 0.56 for ESR and 0.025 for patient disease activity. This discriminant score had an AUC of 0.844 (0.797–0.891) and a sensitivity at the 95% specificity level of 43.8% (95% CI: 38.1–49.2), which is equal to the DAS28 at week 22. The Pearson's correlation coefficient between this discriminant score and the DAS28 was 0.986 (Fig. 2). We also performed logistic regression with similar results (data not shown).

### Comparison with the other DAS scores and SDAI/CDAI

Since different alternative methods are available to calculate the DAS scores (Table 1), we additionally evaluated the properties of those alternative scores. We also evaluated the SDAI and CDAI [8,9], after normalization, by taking the squared root. The Pearson's correlation coefficient of those alternative scores with the DAS28 at week 22 was 0.982 for the DAS28-3, 0.952 for the DAS28-CRP, 0.928 for the DAS28-CRP-3, 0.914 for the SDAI and 0.893 for the CDAI. The AUC and sensitivity at the 95% specificity level are shown in Table 1 and indicate that all those alternative scores perform similarly or slightly worse than the original DAS28.

### Detailed ROC curve analysis of the DAS28

**Figure 3:**
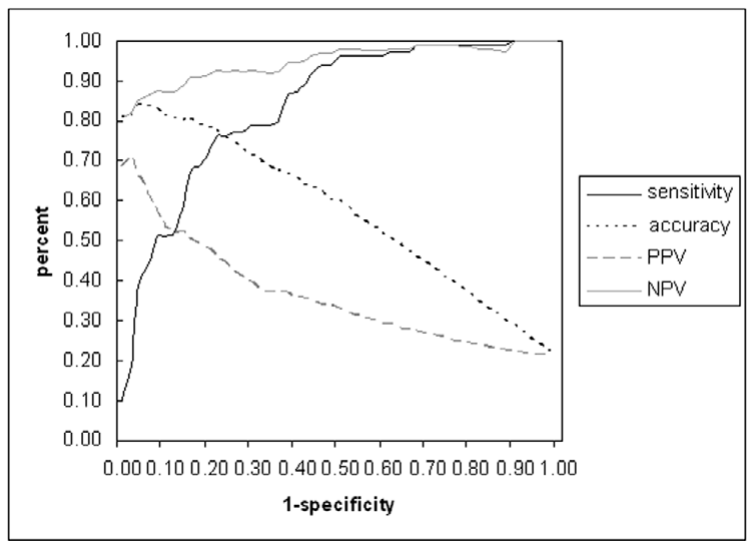
ROC curve analysis of the DAS28 at week 22 (plotting the 1-specificity versus the sensitivity). Also the accuracy, PPV and NPV are plotted. PPV, positive predictive value (predictive value to give a dose increase as a measure of insufficient response); NPV, negative predictive value (predictive value to continue on the current dose as a measure of good response).

We plotted the ROC curve of the DAS28 in Fig. 3 and listed sensitivities and specificities in Table 4. Also, predictive values and the accuracies of classification in function of the different DAS28 cut-offs are shown in Table 4. Beneath a cut-off of 3.2, we found a high predictive value for continuing the current dose as a measure of good response. The maximal accuracy of 84% could be found at a cut-off of 5.5.

## Discussion

The aim of the present analyses was to evaluate which single or composite variables, combined in a model, could discriminate the treating rheumatologist's decision to give a dose increase of infliximab to RA patients not optimally responding to an indicated dose of 3 mg infliximab every 8 weeks. Since different variables on different time points were available, we started to rank the continuous variables based on the AUC of ROC curves and sensitivities at the 95% specificity level. This strategy has previously been proposed for microarray data [17]. The calculation of sensitivities at the 95% specificity level is important in order not to overlook some variables with a relative small AUC but with a high specificity [16]. So, both methods ranked the DAS28 at week 22 as the variable which best discriminates the decision to give a dose increase. In a second and third analysis, we looked at whether response scores and differences in disease activity over time could give additional information to discriminate the rheumatologist's decision. Those analyses indicated that variables, including differences over time, seem to be less important than the momentary remaining disease activity at week 22, to discriminate the rheumatologist's decision.

After the prior selection of variables, based on the findings of the previous steps, we built the final model to discriminate the rheumatologist's decision, which was only slightly better than the DAS28. We think that the small gain in discriminative properties in comparison with the DAS28 is not enough to accept the increased complexity of this model. Moreover, in contrast to the DAS28, this model included the physician's global assessment of disease activity (VAS), which is investigator-dependent and has the draw-back that it cannot be calculated by a study nurse. All four analyses together indicated that the DAS28 is an important variable for evaluating insufficient response to infliximab therapy (especially in daily practice) and that this variable can only slightly be improved by adding supplemental variables.

DAS was developed in the early 1990s [19,20] and later on, it was transformed into the DAS28 [7] in an era when therapy with biologicals was not yet available. In those initial studies, patients were scored by the same two independent nurses and the decision to change disease-modifying antirheumatic drug (DMARD) therapy during a follow-up period of up to 3 years was considered as a measure of insufficient response [20]. The present study is a multi-center study where patients were scored by the treating physician and the decision to give a dose increase of infliximab could happen only at one time point. This difference in study design and therapy may explain why in the present study the AUC of DAS28 is smaller than in other studies (AUC = 0.840 versus 0.933) [21]. Therefore, it is remarkable that despite those differences in study design, we could calculate a discriminant function (in the fifth analysis) that correlated so well with the DAS28 by using the 28SJC, 28TJC, ESR and patient disease activity VAS as independent variables and the physician's decision as a grouping variable. Not only the discriminant scores, but also the coefficients of this discriminant function were quite similar to the coefficients of the DAS28, indicating the robustness of the scores and coefficients of the DAS28 score.

In another, final analysis, we evaluated the alternative DAS scores and the squared root transformed SDAI and CDAI. All those alternative scores have a slightly worse AUC than the original DAS28, but seem good enough to be useful when some other variables are not available. We think the use of the DAS28 is feasible and time-effective using a preprogrammed calculator, spreadsheet or web-based calculator [11]. The unique characteristics of the DAS score make it a useful measure in a lot of applications. DAS28 as a continuous variable is a sensitive tool for measuring response to treatment in randomized controlled trials and facilitates the use of more complex statistical methods that can handle repeated measures over different time points [22-24].

Other studies demonstrated that a low DAS is an important prognostic factor of persistent remission and that DAS correlates with radiological progression [25,26]. DAS may also be a useful parameter in daily clinical practice as a treatment goal and to evaluate the actual disease activity (which cannot be assessed by the categorical response scores) [27-31]. Our findings that the physician's decision to give a dose increase can best be modeled by a combination of measurements of remaining/momentary disease activity, represented by the DAS28 does not reduce the value of the response scores such as ACR response or DAS response scores. Indeed, those scores are important for measuring differences over time as a measure of global treatment effects in clinical trials [12,13] but, as demonstrated by the present study, are not useful for evaluating the momentary disease activity in a single patient, which is important in daily practice. The continuous properties of the DAS28 score provide the additional opportunity for a cut-off, which can be chosen as a function of the purpose. Interestingly, we found a high predictive value for continuing the current dose as a measure of good response below a cut-off of 3.2. It is noteworthy that a DAS score of 3.2 is an important threshold for a good DAS response according to the EULAR criteria [12]. In contrast, for classification purpose, a higher cut-off (5.5) is more appropriate since this level displayed the highest accuracy. One should be aware that the displayed predictive values and accuracies may be highly influenced by the prevalence of insufficient response, reflected by the need for a dose increase, which was 21.5% in the present study. A lower *a priori *chance of the need for a dose increase may increase the accuracy of DAS (given the fixed cut-off of 5.5) and vice versa. Indeed, at a cut-off with a high specificity, the accuracy will increase when the *a priori *chance decreases (applying formula c given in the legend to Table 4).

## Conclusion

The results of the present analyses indicate that the momentary DAS28 as a continuous composite index correlates best with the decision to give a dose increase of infliximab, which is a measure of insufficient response. The discriminative characteristics of the DAS could be slightly improved by the use of supplemental variables, although this results in the disadvantage of a more complex model and calculations. This study also demonstrates the robustness of the scores and coefficients of the DAS28 in a cohort of RA patients under infliximab therapy and therefore validates the DAS28 as a measure of disease activity in patients under treatment with biologicals.

## Abbreviations

ACR = American College of Rheumatology; AUC = area under the curve; CDAI = clinical disease activity score; CI = confidence interval; CRP = C-reactive protein; DAS = disease activity score; DAS28 = Disease Activity Score including a 28-joint count; ESR = erythrocyte sedimentation rate; HAQ = Health Assessment Questionnaire; MTX = methotrexate; RA = rheumatoid arthritis; ROC = receiver-operating characteristic; SDAI = simplified disease activity score; SJC = swollen joint count; SJC28 = 28 swollen joint count; TJC = tender joint count; TJC28 = 28 tender joint count; VAS = visual analogue scale; VAS = Visual Analogue Score.

## Competing interests

AG is an employee of Centocor, Leiden, The Netherlands; NV is an employee of Schering-Plough, Brussels, Belgium; and PD and RW were consultants for Schering-Plough Belgium during the clinical study.

## Authors' contributions

BVC and SVL performed the statistical analysis, constructed the datasets and drafted the manuscript under the direct supervision of LB and FDK. RW, PD, FVdB, EV, HM, LDC, AP, MM, LV and FDK recruited and followed-up the arthritis patients. BVC, SVL, BW, NV, AG, LB, RW, PD and FDK participated in the study design. RW and PD were the initial investigators of the Belgian infliximab expanded-access program, in which the patients were enrolled. All authors have read and approved the final manuscript

## Figures and Tables

**Table 1:** Formulae to calculate the different DAS and SDAI score

Score	Formula	AUC (95% CI)	Sens at 95% spec, % (95% CI)
DAS28	0.56*sqrt(28TJC) + 0.28*sqrt(28SJC) + 0.70*ln(ESR) + 0.014*pt global VAS	0.840 (0.791–0.889)	42.5 (36.9–48.1)
DAS28-3	[0.56*sqrt(28TJC) + 0.28*sqrt(28SJC) + 0.70*ln(ESR)]*1.08 + 0.16	0.815 (0.763–0.868)	37.8 (32.3–43.3)
DAS28-CRP	0.56*sqrt(28TJC) + 0.28*sqrt(28SJC) + 0.36*ln(CRP+1) + 0.014* pt global VAS + 0.96	0.829 (0.782–0.876)	35.8 (30.4–41.2)
DAS28-CRP-3	[0.56*sqrt(28TJC) + 0.28*sqrt(28SJC) + 0.36*ln(CRP+1)] * 1.10 + 1.15	0.806 (0.755–0.858)	28.9 (23.8–33.9)
SDAI	28TJC + 28SJC + CRP/10 + pt global VAS/10 + phys global VAS/10	0.824 (0.776–0.873)	40.7 (35.1–46.2)
CDAI	28TJC + 28SJC + pt global VAS/10 + phys global VAS/10	0.821 (0.772–0.870)	37.8 (32.3–43.2)

DAS28-3 and DAS28-CRP-3 are the DAS28 and DAS28-CRP scores calculated without the patient's global disease activity VAS. AUC, area under the curve; CDAI, clinical disease activity score; CRP, C-reactive protein; DAS, disease activity score; ESR, erythrocyte sedimentation rate; phys, physician; pt, patient; SDAI, simplified disease activity score; SJC28, 28 swollen joint count; TJC28, 28 tender joint count; VAS, Visual Analogue Scale.

**Table 2:** Variables with the highest ranking based on ROC curve AUC and sensitivities at 95% specificity

	AUC	95% CI of AUC
DAS28 w22	0.840	0.791–0.889
28 TJC w22	0.797	0.744–0.850
Physician global VAS w22	0.786	0.736–0.836
Patient pain VAS w22	0.764	0.71–0.814
DAS28 w14	0.750	0.685–0.815
Patient disease activity VAS w22	0.750	0.689–0.802
66TJC w22	0.740	0.689–0.791
28TJC w14	0.721	0.662–0.780
66SJC w22	0.717	0.660–0.774
ESR w22	0.716	0.654–0.779

	Sensitivity (%) at 95% specificity level	95% CI of sensitivity

DAS28 w22	42.5	36.9–48.1
Physician global VAS w22	32.7	28.4–37.0
28SJC w22	29.8	24.7–34.9
Patient pain VAS w22	26.8	22.7–30.9
66SJC w22	24.5	20.6–28.4
ESR w22	24.1	19.7–28.5
66SJC w14	23.0	18.6–27.4
CRP w22	21.3	17.3–25.3
Patient disease activity VAS w22	20.4	16.6-4.2
DAS28 w14	20.3	15.3–25.3

AUC, area under the curve; TJC, tender joint count; SJC, swollen joint count; ESR, erythrocyte sedimentation rate; CI, confidence interval; CRP, C-reactive protein; w, week.

**Table 3:** Sensitivity and specificity of the response scores compared with DAS28 set at equal specificity

	Sensitivity and specificity of the different response scores		Sensitivity of DAS28 at the same specificity level	
	
	Specificity (%)	Sensitivity (%)	Sensitivity (%)	According DAS28 score
No moderate DAS response	83.3	46.7	67.5	4.8
No good DAS response	42.4	96.3	97.3	3.1
No ACR20 response	64.2	69.6	80.0	4.0
No ACR50 response	33.6	85.9	97.5	2.9

DAS, disease activity score.

**Table 4:** Performance at different cut-offs of DAS28 at week 22 for dose increase

DAS cut-off	Sensitivity	Specificity	PPV	NPV	Accuracy
2.0	0.99	0.13	0.23	0.98	0.31
2.5	0.99	0.22	0.25	0.99	0.38
2.6	0.99	0.26	0.26	0.99	0.41
3.0	0.98	0.38	0.29	0.98	0.50
3.2	0.96	0.46	0.32	0.98	0.57
4.0	0.79	0.66	0.38	0.92	0.68
4.5	0.76	0.77	0.47	0.92	0.77
5.0	0.58	0.87	0.55	0.89	0.81
5.1	0.53	0.88	0.55	0.88	0.81
5.5	0.43	0.95	0.69	0.86	0.84
6.0	0.34	0.97	0.73	0.85	0.83
6.5	0.19	0.98	0.72	0.82	0.81

DAS, disease activity score; PPV, positive predictive value (predictive value to give a dose increase as a measure of insufficient response); NPV, negative predictive value (predictive value to continue on the current dose as a measure of good response); PPV, NPV and accuracy were calculated using the following formulae:

c) *Accuracy *= sensitivity* *a*_*priori_chance *+ specificity* (1-*a*_*priori*_*chance*)

The *a priori *chance is given by the percentage of patients that need a dose increase as a measure of insufficient response.

## References

[B1] Lipsky PE, van der Heijde DM, St Clair EW, Furst DE, Breedveld FC, Kalden JR, Smolen JS, Weisman M, Emery P, Feldmann M (2000). Infliximab and methotrexate in the treatment of rheumatoid arthritis. Anti-Tumor Necrosis Factor Trial in Rheumatoid Arthritis with Concomitant Therapy Study Group. N Engl J Med.

[B2] Maini R, St Clair EW, Breedveld F, Furst D, Kalden J, Weisman M, Smolen J, Emery P, Harriman G, Feldmann M, Lipsky P (1999). Infliximab (chimeric anti-tumour necrosis factor alpha monoclonal antibody) versus placebo in rheumatoid arthritis patients receiving concomitant methotrexate: a randomised phase III trial. ATTRACT Study Group. Lancet.

[B3] St Clair EW, Wagner CL, Fasanmade AA, Wang B, Schaible T, Kavanaugh A, Keystone EC (2002). The relationship of serum infliximab concentrations to clinical improvement in rheumatoid arthritis: results from ATTRACT, a multicenter, randomized, double-blind, placebo-controlled trial. Arthritis Rheum.

[B4] Durez P, Van den Bosch F, Corluy L, Veys EM, De Clerck L, Peretz A, Malaise M, Devogelaer JP, Vastesaeger N, Geldhof A, Westhovens R (2005). A dose adjustment in patients with rheumatoid arthritis not optimally responding to a standard dose of infliximab of 3 mg/kg every 8 weeks can be effective: a Belgian prospective study. Rheumatology (Oxford).

[B5] Arnett FC, Edworthy SM, Bloch DA, McShane DJ, Fries JF, Cooper NS, Healey LA, Kaplan SR, Liang MH, Luthra HS (1988). The American Rheumatism Association 1987 revised criteria for the classification of rheumatoid arthritis. Arthritis Rheum.

[B6] Ware JE, Sherbourne CD (1992). The MOS 36-item short-form health survey (SF-36). I. Conceptual framework and item selection. Med Care.

[B7] Prevoo ML, van 't Hof MA, Kuper HH, van Leeuwen MA, van de Putte LB, van Riel PL (1995). Modified disease activity scores that include twenty-eight-joint counts. Development and validation in a prospective longitudinal study of patients with rheumatoid arthritis. Arthritis Rheum.

[B8] Smolen JS, Breedveld FC, Schiff MH, Kalden JR, Emery P, Eberl G, van Riel PL, Tugwell P (2003). A simplified disease activity index for rheumatoid arthritis for use in clinical practice. Rheumatology (Oxford).

[B9] Aletaha D, Nell VPK, Stamm R, Uffmann M, Pflugbeil S, Machold K, Smolen S (2005). Acute phase reactants add little to composite disease activity indices for rheumatoid arthritis: validation of a clinical activitiy score. Arthritis Res Ther.

[B10] Wolfe F (1997). Comparative usefulness of C-reactive protein and erythrocyte sedimentation rate in patients with rheumatoid arthritis. J Rheumatol.

[B11] DAS-Score.NL. http://www.das-score.nl/www.das-score.nl/index.html.

[B12] van Riel PL, van Gestel AM, van de Putte LB (1996). Development and validation of response criteria in rheumatoid arthritis: steps towards an international consensus on prognostic markers. Br J Rheumatol.

[B13] Felson DT, Anderson JJ, Boers M, Bombardier C, Chernoff M, Fried B, Furst D, Goldsmith C, Kieszak S, Lightfoot R (1993). The American College of Rheumatology preliminary core set of disease activity measures for rheumatoid arthritis clinical trials. The Committee on Outcome Measures in Rheumatoid Arthritis Clinical Trials. Arthritis Rheum.

[B14] Harper R, Reeves B (1999). Reporting of precision of estimates for diagnostic accuracy: a review. BMJ.

[B15] Hanley JA, McNeil BJ (1983). A method of comparing the areas under receiver operating characteristic curves derived from the same cases. Radiology.

[B16] Zweig MH, Campbell G (1993). Receiver-operating characteristic (ROC) plots: a fundamental evaluation tool in clinical medicine. Clin Chem.

[B17] Pepe MS, Longton G, Anderson GL, Schummer M (2003). Selecting differentially expressed genes from microarray experiments. Biometrics.

[B18] Hosmer DW, Lemeshow S (2000). Chapter 4. Applied Logistic Regression.

[B19] van der Heijde DM, van 't Hof MA, van Riel PL, Theunisse LA, Lubberts EW, van Leeuwen MA, van Rijswijk MH, van de Putte LB (1990). Judging disease activity in clinical practice in rheumatoid arthritis: first step in the development of a disease activity score. Ann Rheum Dis.

[B20] van der Heijde DM, van 't Hof M, van Riel PL, van de Putte LB (1993). Development of a disease activity score based on judgment in clinical practice by rheumatologists. J Rheumatol.

[B21] Salaffi F, Peroni M, Ferraccioli GF (2000). Discriminating ability of composite indices for measuring disease activity in rheumatoid arthritis: a comparison of the Chronic Arthritis Systemic Index, Disease Activity Score and Thompson's articular index. Rheumatology (Oxford).

[B22] Grigor C, Capell H, Stirling A, McMahon AD, Lock P, Vallance R, Kincaid W, Porter D (2004). Effect of a treatment strategy of tight control for rheumatoid arthritis (the TICORA study): a single-blind randomised controlled trial. Lancet.

[B23] Fuchs HA (1993). The use of the disease activity score in the analysis of clinical trials in rheumatoid arthritis. J Rheumatol.

[B24] Fransen J, van Riel PL (2004). Are better endpoints and better design of clinical trials needed?. Best Pract Res Clin Rheumatol.

[B25] Gossec L, Dougados M, Goupille P, Cantagrel A, Sibilia J, Meyer O, Sany J, Daures JP, Combe B (2004). Prognostic factors for remission in early rheumatoid arthritis: a multiparameter prospective study. Ann Rheum Dis.

[B26] Welsing PM, Landewe RB, van Riel PL, Boers M, van Gestel AM, van der Linden S, Swinkels HL, van der Heijde DM (2004). The relationship between disease activity and radiologic progression in patients with rheumatoid arthritis: a longitudinal analysis. Arthritis Rheum.

[B27] Fransen J, Stucki G, van Riel P (2002). The merits of monitoring: should we follow all our rheumatoid arthritis patients in daily practice?. Rheumatology (Oxford).

[B28] Wolfe F, Cush JJ, O'Dell JR, Kavanaugh A, Kremer JM, Lane NE, Moreland LW, Paulus HE, Pincus T, Russell AS, Wilskie KR (2001). Consensus recommendations for the assessment and treatment of rheumatoid arthritis. J Rheumatol.

[B29] van Riel PL, Schumacher HR (2001). How does one assess early rheumatoid arthritis in daily clinical practice?. Best Pract Res Clin Rheumatol.

[B30] Smolen JS, Aletaha D (2004). Patients with rheumatoid arthritis in clinical care. Ann Rheum Dis.

[B31] Den Broeder AA, Creemers MC, van Gestel AM, van Riel PL (2002). Dose titration using the Disease Activity Score (DAS28) in rheumatoid arthritis patients treated with anti-TNF-alpha. Rheumatology (Oxford).

